# Tribological Properties of DLC Coatings in Model-Based and Real-Life Tests

**DOI:** 10.3390/ma18184251

**Published:** 2025-09-11

**Authors:** Katarzyna Piotrowska, Monika Madej, Krystian Milewski

**Affiliations:** 1 Faculty of Mechatronics and Mechanical Engineering, Kielce University of Technology, Tysiąclecia Państwa Polskiego Ave., 25-614 Kielce, Poland; kpawelec@tu.kielce.pl; 2 TRZUSKAWICA S.A., 26-052 Sitkówka, Poland; krystian.milewski@trzuskawica.pl

**Keywords:** bearings, belt conveyors, friction, DLC coatings, real-life tests

## Abstract

Machinery for internal transport in open-pit mines experiences excessive wear. Belt conveyors used for transporting aggregates are a type of equipment in which bearings are especially prone to failure. Considering the significant financial impact of equipment downtime, ensuring the high reliability of machinery in this sector is paramount. Consequently, the design of tribological interfaces should prioritize maximizing their reliability and minimizing the frequency of malfunctions. This article presents a comparative analysis of 100Cr6 steel and a-C:H type diamond-like carbon (DLC) coatings applied using chemical vapor deposition (PACVD) on bearing components in belt conveyors. Model-based tribological tests were conducted on these materials in both laboratory and real-life settings, evaluating friction and wear under dry friction and under Renolit UNI 3 grease-lubricated conditions, the latter being the operational lubricant for these bearings.

## 1. Introduction

**Figure 1:**
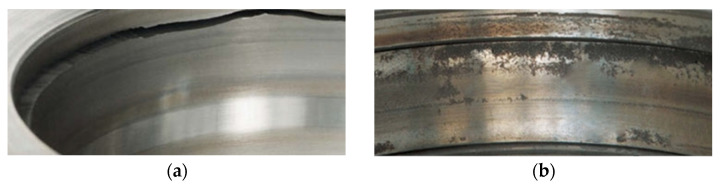
Photographs of the deterioration of rolling bearings used in the aggregate processing industry: (**a**) crack; (**b**) rust.

The aggregate processing industry utilizes a range of mechanical systems, including crushers, screens, apron feeders, pulleys, and belt conveyors, all of which undergo significant operational wear [1]. Bearing elements in conveyors transporting materials over distances ranging from a dozen to several hundred meters are particularly susceptible to wear. Operational conveyor systems are exposed to abrasive stone dust generated from both the processing of raw materials and the transportation of crushed product, as well as to variations in temperature and humidity [2,3,4]. Even with the implementation of different sealing mechanisms, significant amounts of particulate matter from aggregates, prevalent in the operational surroundings, can infiltrate bearing interiors, thereby accelerating their abrasive degradation. The degradation of machinery components due to corrosion from contact with rainwater or process water used for aggregate rinsing presents a considerable risk. Furthermore, inadequate or incorrect lubrication contributes to bearing failure by increasing friction and causing overheating, which subsequently leads to pitting on the rolling surfaces and a reduction in operational lifespan. Vibrations and dynamic loads, particularly those caused by uneven conveyor loading or the transportation of course materials, also pose challenges. Extended exposure to these detrimental factors leads to the development of microcracks and material fatigue, resulting in machinery and equipment within the aggregate industry experiencing both tribological wear and other modes of deterioration [5,6,7,8,9]. Principally, each failure substantially contributes to increased production costs. Figure 1 shows two types of damage in rolling bearings used in the aggregate industry.

The adverse impacts of friction can be mitigated through the use of specialized lubricants and highly abrasion-resistant surface layers. Specifically chosen to meet the particular operational demands of a tribological system, they aim to reduce the friction coefficient, increase viscosity, enhance lubrication properties, and improve corrosion resistance [10].

The search for innovative solutions, driven by the rising costs of operating and maintaining industrial machinery and equipment, is a major factor in efforts to tailor diamond-like carbon coating properties for the harsh conditions typical in aggregate processing [11]. This adaptation has the potential to significantly improve the lifespan and reliability of machine components, which is vital for optimizing operational costs and enhancing the effectiveness of industrial procedures. Equipment breakdowns leading to production halts significantly impact a company’s financial standing and reputation, particularly through customer order delays [12,13]. Consequently, the careful design of advanced tribological interfaces is crucial, especially for the aggregate sector, necessitating the selection of suitable anti-wear coatings and lubricants for specific friction point operating conditions. Furthermore, prior to field testing, it is essential to conduct appropriate laboratory experiments to provide an initial assessment of the effectiveness of the implemented solutions.

The materials and lubricants currently used in the working parts of machinery often fail to meet the growing demands of the aggregate processing industry, which operates under extreme conditions. Consequently, ongoing progress is essential through the development of more robust alloys, advanced protective coatings, and high-performance lubricants. This will lead to increased equipment lifespan and efficiency, while also reducing operational and maintenance expenses.

Anti-wear surface layers and coatings, such as diamond-like carbon (DLC) coatings applied in both lubricated and unlubricated friction nodes [8,14], have gained significant attention. High hardness and wear resistance, as well as a low coefficient of friction, make these coatings an exceptionally effective solution under conditions of intensive operation [15,16]. Their fundamental properties, mechanisms of action, and applications have been comprehensively reviewed in traditional works [17], while more recent reviews highlight the role of surface chemistry, hydrogen content, and tribochemical processes in achieving superlubricity in DLC films [18].

In [19], the authors compared the tribological properties of black iron oxide and WC/a-C:H coatings deposited on rolling bearings. The tests were performed on rolling bearings under laboratory conditions simulating environments with limited lubrication (which is typical in real mechanical applications). The obtained results indicated that WC/a-C:H coatings are more effective in protecting against adhesive wear and enhancing the operational durability of bearings under boundary lubrication conditions compared to the results for black oxide coatings. Additionally. WC/a-C:H coatings exhibited significantly lower wear and friction coefficients than those of black oxide. The authors of reference [20] analyzed the tribological behavior of ball bearings with a DLC coating deposited on both the inner and outer rings, focusing on the ball material and the effect of lubrication. The research outcomes indicated that pairing an a-C:H:Me coating with Si_3_N_4_ balls provided the most favorable performance, characterized by a consistent friction force throughout the duration of the experiment.

Furthermore, the use of DLC coatings in ball bearings eliminates the need for traditional mineral-oil-based lubricants, thereby contributing to energy and resource savings and enhancing the durability of bearings under dry friction conditions. The above studies provide valuable information on the potential applications of DLC coatings in industrial settings. The authors of [21] analyzed the influence of various gear oils on the wear of DLC coatings in gear applications. The research aimed to evaluate the effectiveness of the coatings under conditions similar to real-life conditions, taking into account various types of lubrication. The results of these studies indicate that the W-DLC/CrN coating effectively reduces abrasive wear and scuffing, particularly when used with PAO oil. However, it failed to improve pitting resistance, suggesting the need for further research on optimizing coatings for this type of damage.

DLC coatings were also investigated in the context of their application on rolling bearing components in the aerospace industry [22,23]. Pure hydrogenated DLC coatings were deposited on AISI 52100 steel. The authors aimed for high wear resistance coatings and a low coefficient of friction, both in vacuum and normal atmospheric environments. The tribological behavior was assessed in varying test environments (air, vacuum, and nitrogen) using a ball-on-disc tribometer, which employed AISI 52100 steel counter bodies. The measured mean coefficients of friction were in the ranges of 0.22–0.27 (in air), 0.02–0.03 (in nitrogen), and 0.007–0.013 (in vacuum). These results indicate a significant reduction in motion resistance achieved by the DLC coatings, especially in vacuum, positioning them as a potential material for tribological applications.

A comprehensive analysis of the effect of DLC coatings on the strength of 18CrNiMo7–6 steel gear teeth is presented in [24]. The study focused on strength and scuffing resistance tests. The results demonstrated that the DLC coating increased the surface hardness to approximately 2000 HV and the critical scuffing temperature from 224.6 °C to 348.6 °C, indicating improved resistance to extreme operating conditions. The findings of these studies suggest that DLC coatings significantly enhance the durability and reliability of machine components subjected to high loads and extreme temperatures. This is particularly important in the automotive, aerospace, and heavy machinery sectors, where mechanical components are intensely exposed to severe wear.

Recent research on the use of solid lubricants in combination with DLC has demonstrated that these solutions significantly improve the durability of the surface layer and enable the achievement of low friction coefficients in conditions of limited access to lubricating oils. Recent publications confirm this potential, demonstrating that appropriate functionalization of DLC with solid materials such as graphene, nanodiamonds, or hBN leads to the formation of stable transfer layers and enables the achievement of ultra-low friction—under both dry (<0.05) and humid (<0.1) conditions. However, some of the described methods require specific conditions, such as heat treatment in a vacuum. This clearly indicates that the integration of DLC with advanced solid lubricants is a promising development path in tribology [25,26,27,28,29].

Despite the significant interest in DLC coatings, the existing literature still lacks detailed studies regarding their application on aggregate processing equipment considering the specific operating conditions involving high abrasion, variable loads, and intensive wear. Most available research focuses on the use of DLC coatings in bearings, where their properties enable improved wear resistance, enhanced lubrication efficiency, and reduced energy losses. The aim of this work was to conduct a comparative analysis of how diamond-like carbon coatings influence the operational durability of bearings and to explore their potential application in belt conveyors.

## 2. Materials and Methods

**Figure 2:**
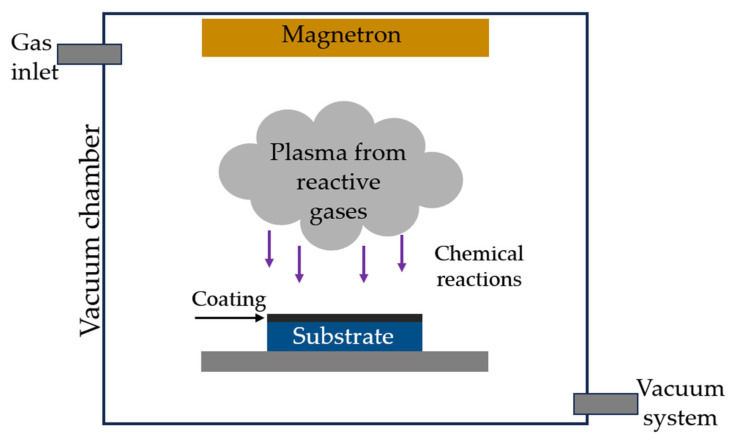
Schematic of the DLC coating deposition process—PACVD.

The study investigated DLC coatings deposited on 100Cr6 steel using plasma-assisted chemical vapor deposition (PACVD) [30,31,32] with a glow discharge at a temperature below 250 °C. The layers were made using an RS 50 (Oerlikon Balzers, Pfäffikon, Switzerland) coating system. The selection of the above DLC coatings was based on the positive results of previous studies [1,6,7,11]. A schematic of the process is shown in Figure 2.

The thickness of the deposited coatings was examined via microscopic observations of the cross-sections using a JSM 7100F scanning electron microscope (JEOL, Tokyo, Japan) equipped with an EDS energy-dispersion spectrometer. An accelerating voltage of 15 kV and a magnification of ×20,000 were used. The study was extended to include point analyses of elemental composition in micro-areas. The test results are presented in Section 3.1.

**Figure 3:**
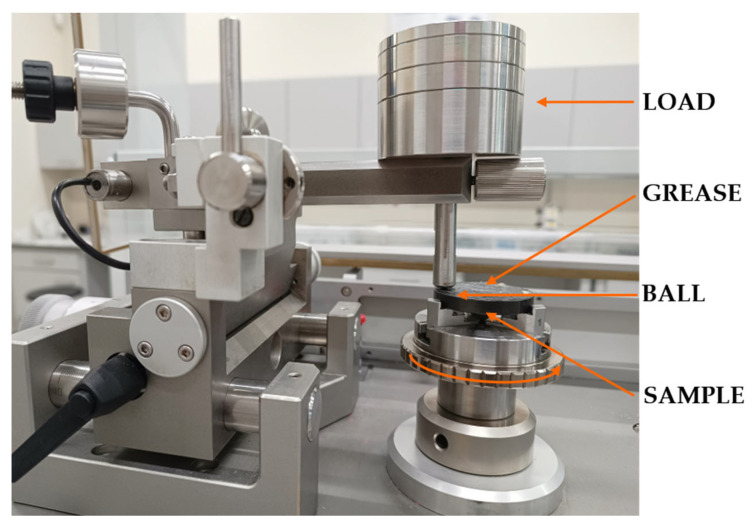
Friction pair.

Model-based tribotests were performed on both coated (a-C:H) and uncoated 100Cr6 steel discs (38 mm diameter, 6 mm height). The elemental composition of the 100Cr6 steel is detailed in Table 1. A TRB^3^ tribometer (Anton Paar, Baden, Switzerland) was used in a rotary configuration under both dry and lubricated friction conditions. The test parameters are shown in Table 2, and the friction pair is shown in Figure 3.

The samples were subjected to a normal load—F_N_—of 10 N and 50 N, corresponding to the estimated initial contact pressures of around 1420 GPa and 2430 GPa for 100Cr6 and 1610 GPa and 2755 GPa for DLC.

For the lubricated tests, Renolit UNI 3 grease was used, a general-purpose grease composed of highly refined mineral oil and a lithium soap thickener (12-hydroxystearic acid), with selected properties listed in Table 3. During the tests, a layer of grease approximately 1 mm thick was applied to the sample. Following the tribological testing, the surface topography of the contact areas was analyzed. The results of the experiments are presented in Section 3.2.

Observations of the surface geometrical structure after the model-based tests were performed using a Leica DCM8 non-contact profilometer (Leica, Heerbrugg, Switzerland). The analysis of the test results was carried out using LeicaMap 7.3 software. A 20× objective lens was used to characterize the operational surface layer. For each sample, a series of five measurements of a 1.2 mm × 1.2 mm area were captured in confocal mode. The surface topography analysis was conducted based on micrographs, 3D axonometric images, average transverse profiles of wear tracks, and the volumetric wear rate. The results of the experiment are presented in Section 3.3.

An additional experiment was conducted with DLC coating on a real bearing in service. The wear tracks on the outer raceway of the bearings, observed after tests under real-life conditions, were measured using a Form Talysurf PGI 1230 contact profilometer (Taylor Hobson, AMETEK, Inc., Berwyn, PA, USA). Due to the curved surface geometry of the bearings, which resulted in a large number of unmeasured points in the non-contact measurements, the contact method was employed to enable accurate mapping of the surface topography. Based on the obtained results, 3D axonometric images, average profiles of the wear tracks generated based on 50 profiles, and the most important amplitude parameters were prepared according to ISO 21920-2 [33]. The results are presented in Section 3.4.

## 3. Results and Discussion

### 3.1. Evaluation of DLC Coating Microstructure

**Figure 4:**
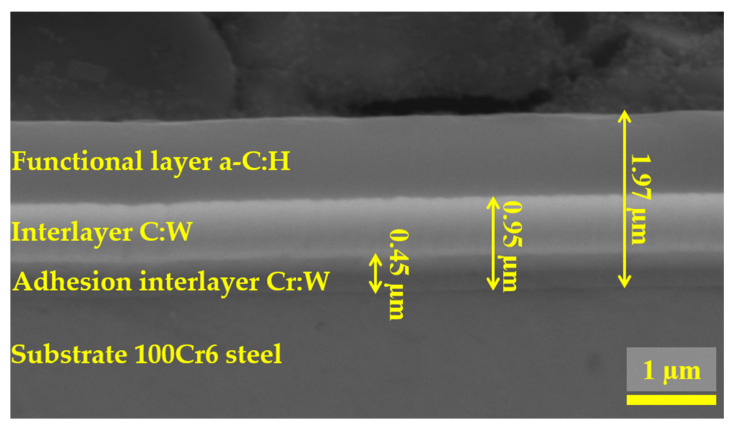
Morphology of the cross-section of the DLC coating.

**Figure 5:**
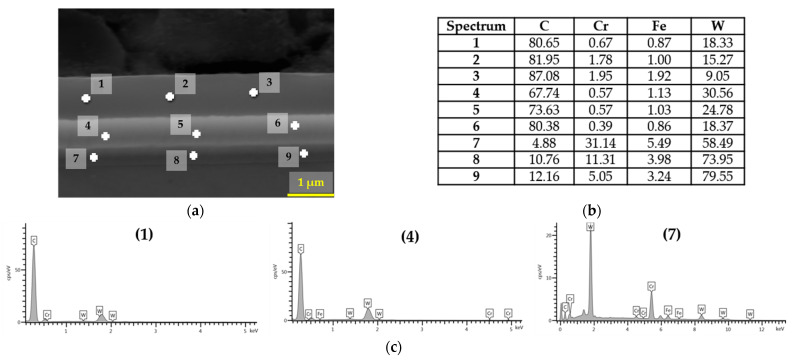
EDS analysis in micro-areas: (**a**) micrograph; (**b**) wt%; (**c**) characteristic radiation spectrum at points 1, 4, and 7.

**Figure 6:**
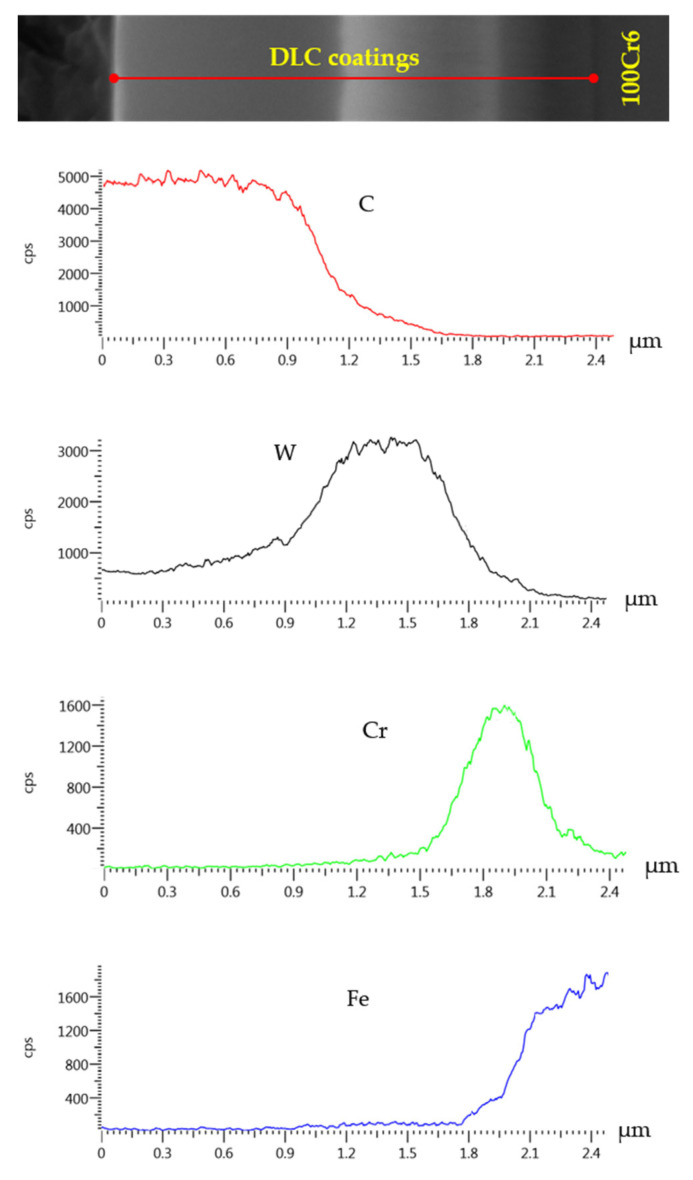
Linear distribution of elements on the cross-section.

Figure 4, Figure 5 and Figure 6 present the morphology of the DLC coating. Specifically, Figure 4 illustrates the coating thickness, Figure 5 presents the chemical composition in selected micro-regions, and Figure 6 shows the linear elemental distribution.

Microstructure analysis revealed that the total coating thickness was approximately 2 µm, exhibiting a homogeneous structure across the entire examined surface. This suggests a stable deposition process and uniform coverage of the substrate, 100Cr6 steel. The homogeneity of the layer is significant from the perspective of its mechanical and tribological properties, as it minimizes the risk of localized stress concentrations that could lead to premature wear or delamination of the coating during operation.

The EDS analysis of the a-C:H coating in micro-areas revealed that the near-surface layer consisted of carbon and tungsten, while a chromium layer, acting as an interlayer, was identified at a depth of approximately 1.5 µm from the surface. The use of chromium was aimed at improving the adhesion of the coating to the metallic substrate.

### 3.2. Model-Based Tribological Tests

**Figure 7:**
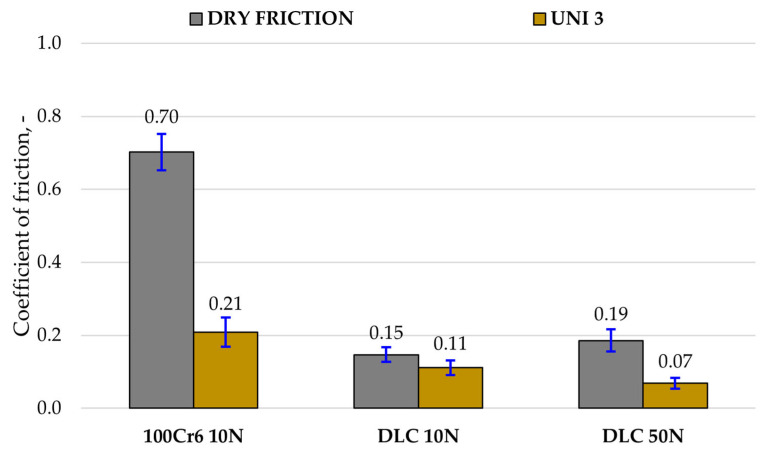
Mean values of friction coefficients obtained in model-based tests.

**Figure 8:**
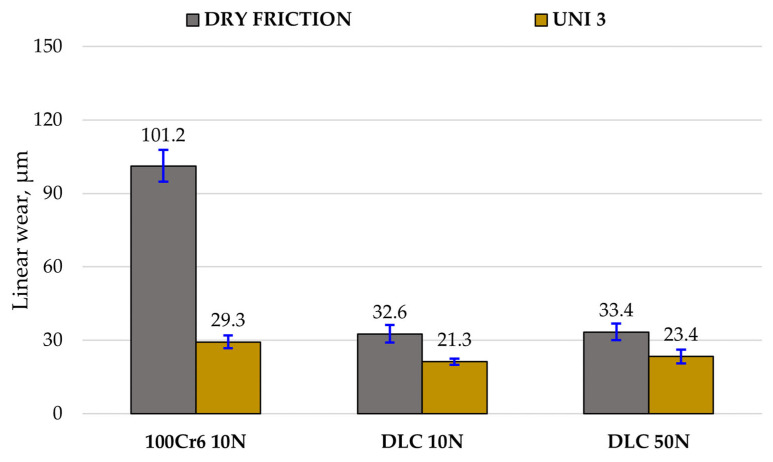
Mean linear wear of the tribological pairs obtained in model-based tests.

Figure 7 and Figure 8 present the results of model-based tribotests conducted under dry (DF) and lubricated friction conditions using Renolit UNI 3 grease (UNI 3). The values of the friction coefficient and linear wear of the tested tribological pairs were recorded during the tests. Mean values and standard deviation of these parameters were determined based on three measurement series.

The deposition of a DLC coating enhanced the tribological performance of 100Cr6 steel under both 10 N and 50 N loads. Furthermore, lubrication improved operating conditions for both uncoated steel and the DLC-coated samples, with the most substantial benefits observed when the DLC coating and lubrication were combined.

Specifically, at a 10 N load, the DLC coating reduced the friction coefficient by nearly fivefold under technically dry conditions (0.70 to 0.15) and almost twofold with lubrication (0.21 to 0.11) when compared to the results for uncoated steel. Notably, tribological wear tests on the uncoated 100Cr6 steel–steel friction node at 50 N were not feasible due to a rapid increase in friction force (exceeding 25 N) that activated overload protection in the tribometer; this occurred during all three attempts.

However, tests on the DLC-coated samples at 50 N showed a reduction in motion resistance. Under dry conditions, the friction coefficient for the DLC coating increased slightly from 0.15 (at 10 N) to 0.19 (at 50 N). Still, with lubrication, it decreased significantly from 0.11 to 0.07, highlighting a synergistic effect with the lubricant, promoting low-shear sliding. Linear wear measurements confirmed that the DLC coating reduced the linear wear of 100Cr6 steel by approximately 68% under technically dry conditions and about 27% with lubrication. The results confirm that the DLC coating maintains low wear rates even under increased load.

The results show that at low load (10 N), lubrication significantly reduces the wear of 100Cr6 steel, while the DLC coating already provides low wear in dry conditions and exhibits an additional, although smaller, benefit with lubrication. At higher load (50 N), DLC wear remains nearly unchanged under both dry and lubricated conditions, demonstrating the coating’s stability and resistance to load increase.

In conclusion, the combination of a DLC coating and lubrication yielded the best results for the tested tribopairs, achieving the lowest friction coefficients and linear wear. This suggests their promising potential as anti-wear solutions.

### 3.3. Tribological Wear Analysis Following the Model-Based Tests

After the model-based tests, the resulting wear tracks were subjected to microscopic observations. Photographs, 3D axonometric images, transverse profiles of the wear tracks, and volumetric wear rates calculated according to the following formula were analyzed [34]:

(1) $$W_{V}=\frac{V}{F_{N}*s}$$

where:

V—volume of material removed, mm^3;^
$F_{N}$—normal force, N;
s—distance, m.

**Figure 9:**
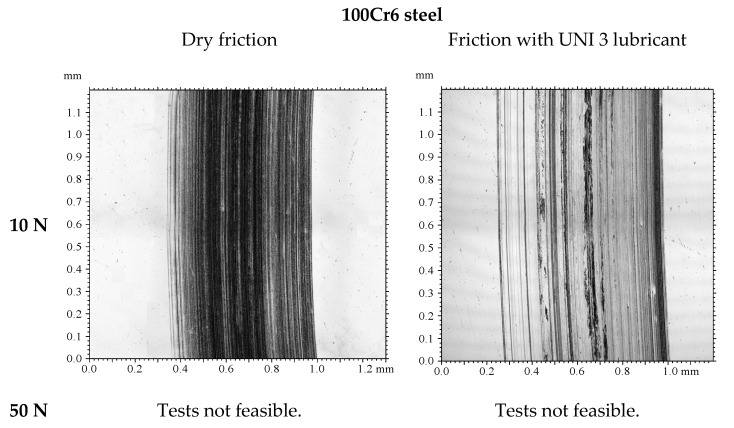
Photographs of wear tracks on 100Cr6 steel.

**Figure 10:**
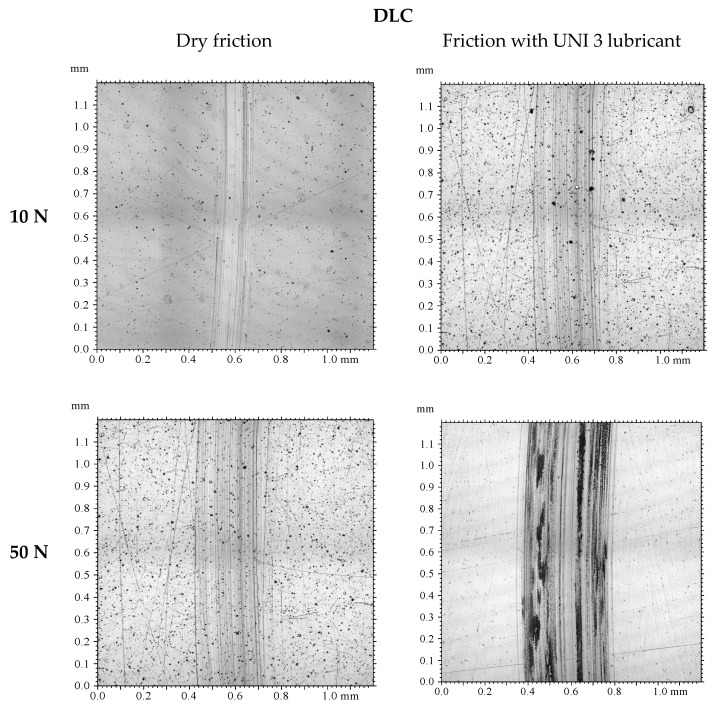
Photographs of wear tracks on the DLC coating.

**Figure 11:**
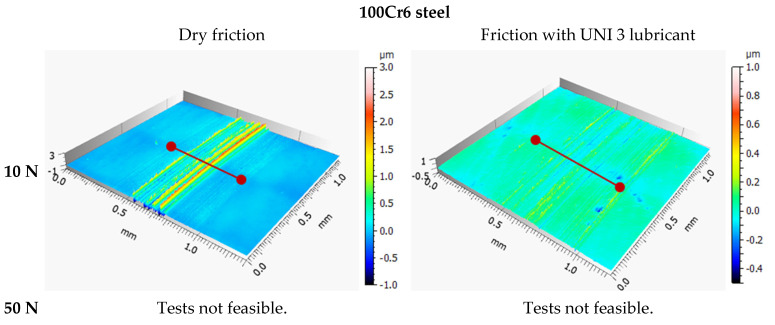
Axonometric views of wear tracks on 100Cr6 steel.

**Figure 12:**
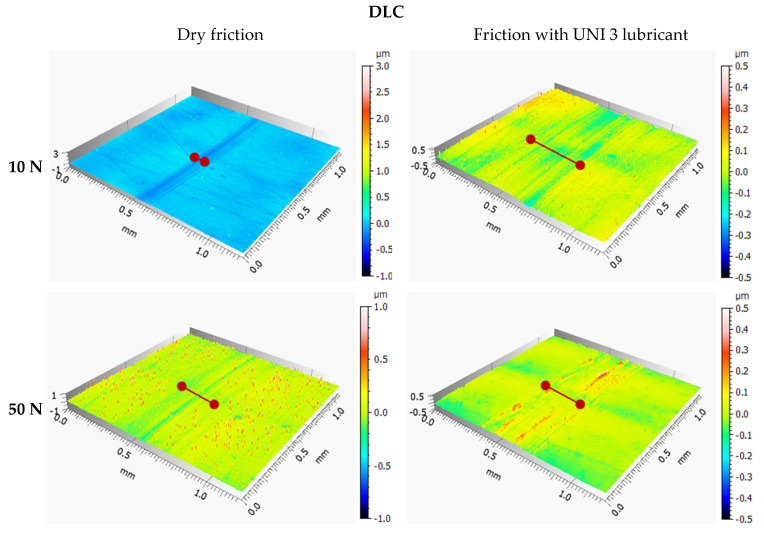
Axonometric views of wear tracks on the DLC coating.

**Figure 13:**
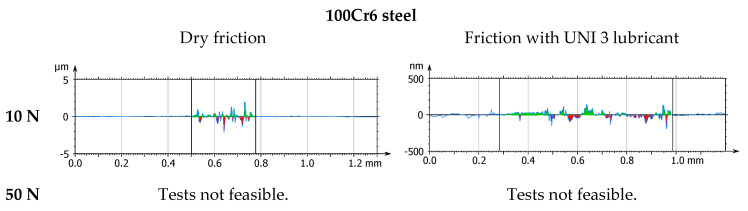
Transverse profiles of wear tracks on 100Cr6 steel.

**Figure 14:**
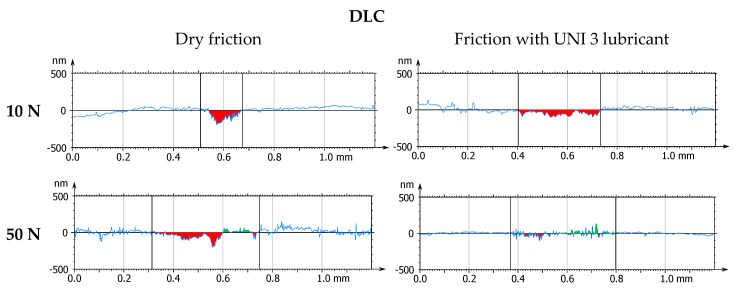
Transverse profiles of wear tracks on the DLC coating.

The results are shown in Figure 9, Figure 10, Figure 11, Figure 12, Figure 13 and Figure 14 and Table 4.

Analysis of sample wear following the model-based tests reveals that applying a DLC coating significantly decreased volumetric wear by 87% under technically dry conditions and by 77% with lubrication. Lubrication was most effective for the DLC coating at both 10 N and 50 N loads. Notably, the volumetric wear of the DLC coating remained relatively consistent when the load increased from 10 N to 50 N. While lubrication reduced wear for both 100Cr6 steel and DLC-coated samples, the effect was more pronounced for the DLC coating. These findings demonstrate that DLC coatings possess high resistance to increased loads and provide consistently low wear rates, positioning them as an excellent choice for components prone to excessive wear.

### 3.4. Tribological Tests—Real-Life Conditions

For the real-life tests, a self-aligning ball bearing with a tapered bore, model 1217 K (ISO designation) from SKF (Svenska Kullagerfabriken，Poznań，Poland), was used. These bearings feature two rows of balls, a common spherical raceway in the outer ring, and two deep, uninterrupted raceway grooves in the inner ring. It has a tapered bore (suffix “K”) and is supplied with normal radial internal clearance (C_0_/C_N_). Their design makes them insensitive to angular misalignment between the shaft and the housing, which can be caused by, for example, shaft deflection. The tapered bore allows for easy bearing mounting using an adapter or withdrawal sleeves. The dimensions are: 85 mm bore (d), 150 mm outside diameter (D), 28 mm width (B), shoulder diameter inner ring (d1), shoulder diameter outer ring (D_1_) and min. 2 mm chamfer dimension (r_1,2_). It is unsealed (no-seal) and uses a steel cage. The basic dynamic load rating (C) is approximately 48.8 kN, and the basic static load rating (C_0_) is approximately 20.8 kN. The reference limiting speed is around 4700 rpm [35].

**Figure 15:**
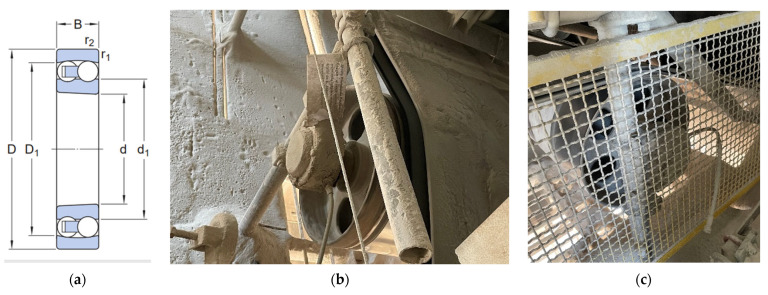
Technical drawing of the bearing used for tests under real-life conditions [35] (**a**); photographs of bearing assembly locations (**b**,**c**).

Components with and without DLC coatings were installed in the existing bearing housings of belt conveyors used for aggregate transport at the Aggregate Production and Dispatch Department of Trzuskawica S.A. Due to significant mechanical loads, intense material disintegration, and unfavorable environmental conditions—including corrosive effects—the operating conditions of these devices are classified as particularly harsh. Figure 15 shows a view of the bearing used for the real-life tests and its mounting location. It was assumed that, in the event of failure or after the end of their service life, the bearings were dismounted and submitted for technical evaluation and wear analysis.

### 3.5. Tribological Wear Analysis After Real-Life Tests

**Figure 16:**
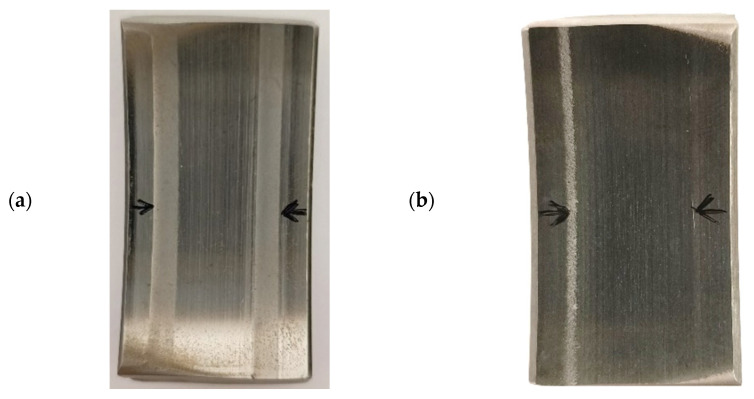
Photographs of bearing raceway segments after real-life condition tests: (**a**) without coating; (**b**) with a DLC coating.

**Figure 17:**
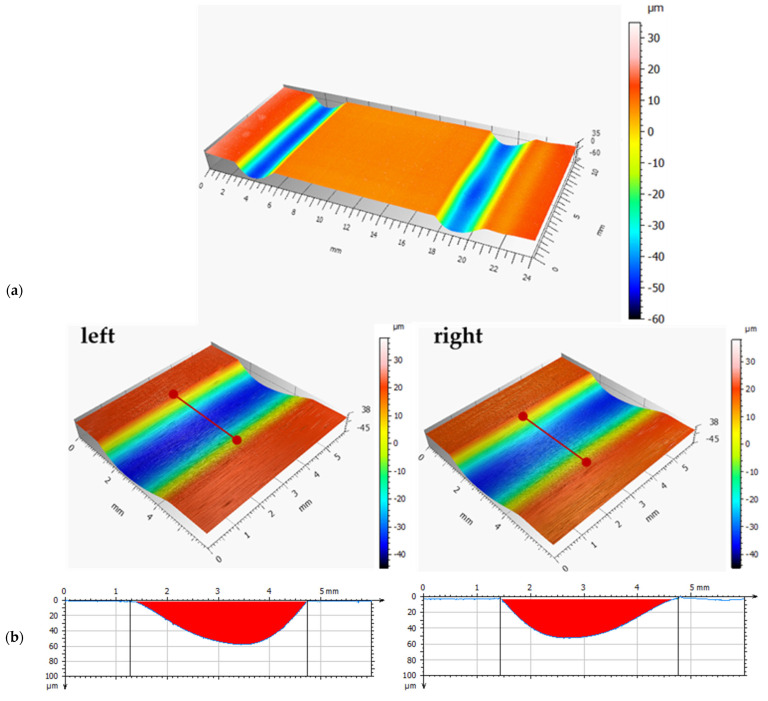
Wear analysis of a bearing without the coating: (**a**) 3D axonometric image; (**b**) wear profile.

**Figure 18:**
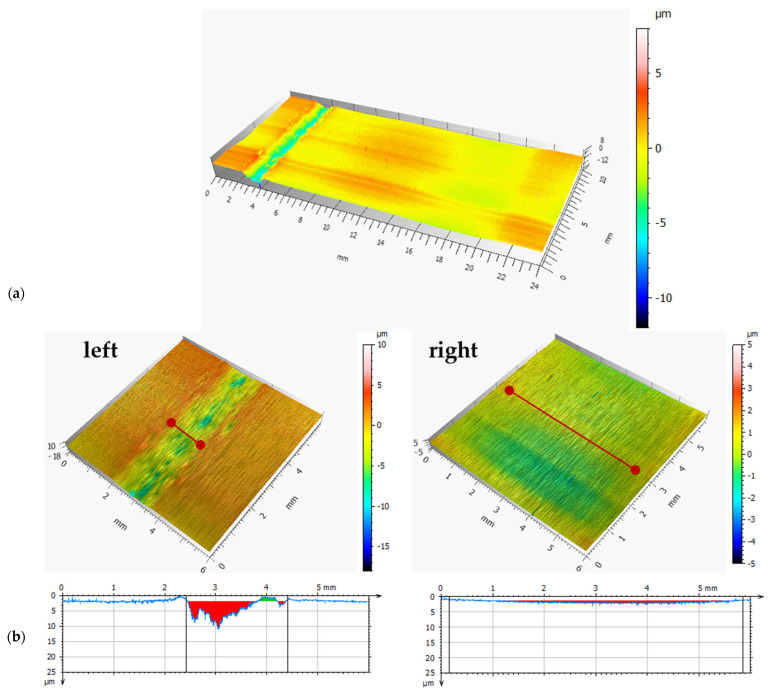
Wear analysis of a bearing with the DLC coating: (**a**) 3D axonometric image; (**b**) wear profile.

Figure 16 shows photographs of bearing segments after real-life tests: without coating (a), with a DLC coating (b). Figure 17 and Figure 18 present the results of microscopic observation studies after real-life tests performed using contact profilometry. The 3D axonometric images (a) and wear track profiles in the cross-section (b) are compared. The wear analysis of bearings with and without a DLC coating was carried out based on three wear parameters: maximum wear track depth, cross-sectional area, and wear track volume (Table 5).

After just six months of operation, the uncoated bearing failed and required replacement. However, the DLC-coated bearing continued to operate stably, showing no signs of critical damage. After 18 months, the decision was made to disassemble the bearing and conduct a wear assessment to verify the coating’s actual effectiveness under industrial conditions.

The research results obtained within the scope of this work indicate that DLC coatings applied to the outer raceways of bearings contribute to the improvement of the tribological properties of the bearings, particularly in terms of reducing the friction coefficient and wear. This finding is confirmed by shallower wear track depths, smaller surface area, lower volumetric wear, and over three times longer operational life of the bearings with the DLC coating. Extending the service life of friction components in belt conveyors increases the operational availability of these machines in the aggregate industry.

## 4. Conclusions

Model tests provide wear data, such as the coefficient of friction and volumetric wear, under controlled loads, lubrication times, and conditions. These results allow us to determine how the DLC coating reduces friction and wear compared to the results for uncoated steel. However, they do not directly predict bearing service life. The relationship between volumetric wear under laboratory conditions and bearing service life under real-world conditions is therefore qualitative, not quantitative. The laboratory tests with simplified contact geometry and controlled loads (10 and 50 N) demonstrate a low coefficient of friction and high wear resistance of the DLC coating under both dry friction and UNI 3 grease lubrication. Real-life tests take into account additional factors: dynamic loading, vibration, grease contamination, temperature gradients, and longer operating periods. These factors can lead to fatigue wear, pitting, and microcracks in the bearings. These conditions are not addressed in short-term tribological tests conducted in the laboratory.

Overall, laboratory tests demonstrate the potential of DLC coatings to reduce friction and wear, making them an exceptionally promising solution for bearing applications, but translating this into extended bearing service life requires additional, long-term testing.

## Figures and Tables

**Table 1:** Chemical composition of 100Cr6 steel/wt. %.

Element	C	Mn	Si	P	S	Cr
Value	0.95–1.10	0.20–0.50	max–0.35	max–0.025	1.30–1.60	1.30–1.60

**Table 2:** Tribological test parameters.

Parameter	Value and Unit
Type of movement	Rotary
Load	10 N and 50 N
Linear speed	0.1 m/s
Distance	1000 m
Friction radius	12 mm
Ambient temperature	23 ± 1 °C
Humidity	50 ± 1%
Lubricant	Without (DF), UNI 3
Lubricant temperature	23 ± 1 °C
Counter sample	100Cr6 (Ø 6 mm)

**Table 3:** Selected properties of Renolit UNI 3 grease.

Property	Unit	Value
Color	-	Amber
Dropping point	°C	>190
Viscosity at 40 °C	mm^2^/s	100
Operating temperature range	°C	−30 ÷ +130

**Table 4:** Volumetric wear rates.

	Volumetric Wear Rate, $\left[\frac{{\mathrm{mm}}^{3}}{N\times m}\right]$	
	10 N	50 N
100Cr6 DF	$1.4\times 10^{-8}$	-
100Cr6 UNI 3	$3.9\times 10^{-9}$	-
DLC DF	$1.8\times 10^{-9}$	$2.0\times 10^{-9}$
DLC UNI 3	$8.9\times 10^{-10}$	$1.8\times 10^{-9}$

**Table 5:** Bearing wear parameters.

		Max. Depth, mm	Surface Area, mm^2^	Volume, mm^3^
100Cr6	left	0.057	0.122	0.6498
	right	0.050	0.102	0.532
DLC	left	0.009	0.0057	0.0338
	right	0.001	0.0042	0.2180

## Data Availability

The original contributions presented in this study are included in the article. Further inquiries can be directed to the corresponding author.
